# Homocysteine-Lowering by B Vitamins Slows the Rate of Accelerated Brain Atrophy in Mild Cognitive Impairment: A Randomized Controlled Trial

**DOI:** 10.1371/journal.pone.0012244

**Published:** 2010-09-08

**Authors:** A. David Smith, Stephen M. Smith, Celeste A. de Jager, Philippa Whitbread, Carole Johnston, Grzegorz Agacinski, Abderrahim Oulhaj, Kevin M. Bradley, Robin Jacoby, Helga Refsum

**Affiliations:** 1 Oxford Project to Investigate Memory and Ageing (OPTIMA), University of Oxford, Oxford, United Kingdom; 2 University Department of Pharmacology and Department of Physiology, Anatomy & Genetics, University of Oxford, Oxford, United Kingdom; 3 Department of Clinical Neurology, Oxford Centre for Functional Magnetic Resonance Imaging of the Brain, University of Oxford, Oxford, United Kingdom; 4 Department of Radiology and Nuclear Medicine, Oxford Radcliffe Hospitals NHS Trust, Oxford, United Kingdom; 5 University Department of Psychiatry, University of Oxford, Oxford, United Kingdom; 6 Department of Nutrition, Institute of Basic Medical Sciences, University of Oslo, Oslo, Norway; Mental Health Research Institute of Victoria, Australia

## Abstract

**Background:**

An increased rate of brain atrophy is often observed in older subjects, in particular those who suffer from cognitive decline. Homocysteine is a risk factor for brain atrophy, cognitive impairment and dementia. Plasma concentrations of homocysteine can be lowered by dietary administration of B vitamins.

**Objective:**

To determine whether supplementation with B vitamins that lower levels of plasma total homocysteine can slow the rate of brain atrophy in subjects with mild cognitive impairment in a randomised controlled trial (VITACOG, ISRCTN 94410159).

**Methods and Findings:**

Single-center, randomized, double-blind controlled trial of high-dose folic acid, vitamins B_6_ and B_12_ in 271 individuals (of 646 screened) over 70 y old with mild cognitive impairment. A subset (187) volunteered to have cranial MRI scans at the start and finish of the study. Participants were randomly assigned to two groups of equal size, one treated with folic acid (0.8 mg/d), vitamin B_12_ (0.5 mg/d) and vitamin B_6_ (20 mg/d), the other with placebo; treatment was for 24 months. The main outcome measure was the change in the rate of atrophy of the whole brain assessed by serial volumetric MRI scans.

**Results:**

A total of 168 participants (85 in active treatment group; 83 receiving placebo) completed the MRI section of the trial. The mean rate of brain atrophy per year was 0.76% [95% CI, 0.63–0.90] in the active treatment group and 1.08% [0.94–1.22] in the placebo group (*P* = 0.001). The treatment response was related to baseline homocysteine levels: the rate of atrophy in participants with homocysteine >13 µmol/L was 53% lower in the active treatment group (*P* = 0.001). A greater rate of atrophy was associated with a lower final cognitive test scores. There was no difference in serious adverse events according to treatment category.

**Conclusions and Significance:**

The accelerated rate of brain atrophy in elderly with mild cognitive impairment can be slowed by treatment with homocysteine-lowering B vitamins. Sixteen percent of those over 70 y old have mild cognitive impairment and half of these develop Alzheimer's disease. Since accelerated brain atrophy is a characteristic of subjects with mild cognitive impairment who convert to Alzheimer's disease, trials are needed to see if the same treatment will delay the development of Alzheimer's disease.

**Trial Registration:**

Controlled-Trials.com ISRCTN94410159

## Introduction

In elderly, the brain shows significant progressive atrophy. The atrophy occurs even in cognitively healthy subjects [1] but is much accelerated in patients suffering from Alzheimer's disease [2], [3], [4], [5]. An intermediate rate of atrophy is found in people with mild cognitive impairment (MCI) [6], [7], [8], [9], [10], [11]. Since the rate of brain atrophy is more rapid in subjects with MCI who convert to Alzheimer's disease [5], it is important to identify factors that determine the rate of atrophy since reducing the rate of atrophy is likely to slow the conversion to Alzheimer's disease. One such factor appears to be raised concentrations of plasma total homocysteine (tHcy). Moderately elevated concentrations of tHcy have been associated with an increased risk of dementia, notably Alzheimer's disease, in many cross-sectional and prospective studies [12], [13], [14], [15], [16]. Raised tHcy is also associated with both regional and whole brain atrophy, not only in Alzheimer's disease [12] but also in healthy elderly [17], [18], [19], [20], [21].

The tissue and plasma concentrations of homocysteine are largely determined by the body's status of certain B vitamins (folate, B6 and B12), which are cofactors or substrates for enzymes involved in homocysteine metabolism [22]. The VITACOG trial reported here was designed to see if lowering tHcy concentrations by the administration of high doses of supplementary B vitamins (folic acid, vitamins B_6_ and B_12_) over two years would slow the rate of atrophy of the brain in elderly subjects with MCI. This group was chosen because they suffer from modestly increased rate of atrophy, making it possible to detect significant changes in rate of atrophy in a relatively small number of subjects followed for relatively short time. The chosen doses of vitamins lower tHcy levels by about 30% in populations from countries without mandatory folic acid fortification of flour [23].

## Methods

The study was carried out according to the principles expressed in the Declaration of Helsinki and was approved by a local NHS research ethics committee (COREC 04/Q1604/100). Each subject gave written consent for their participation. The Protocol for this Trial and supporting CONSORT checklist are available as supporting information; see Protocol S1 and Checklist S1.

### Study protocol

Participants in the Oxford area were recruited between April 2004 and November 2006 through advertisements in the local newspaper or radio seeking elderly people with concerns about their memory. The trial has been registered as VITACOG, ‘Homocysteine and B vitamins in cognitive impairment’ ISRCTN 94410159 (http://www.controlled-trials.com/).

Inclusion criteria included: age ≥70 years; study partner available as informant, and diagnosis of amnestic or non-amnestic MCI according to Petersen's criteria [24]. The diagnosis included a subjective concern about memory that did not interfere with activities of daily living, assessed with 4 questions on subjective memory complaints from the Cambridge Mental Disorders of the Elderly Examination (CAMDEX) [25] and 5 questions relating to activities of daily living based on the Cambridge Behavioural Inventory [26], an objective memory problem assessed with the ‘Telephone interview of cognitive status, modified’ (TICS-M) [27], a test without a ceiling effect [28], and category fluency [29] based on previously defined cut-off scores for MCI [28]. Thus, eligible subjects had a score of 17 – 29 out of a maximum of 39 on TICS-M. For borderline cases, if TICS-M was >29 but category fluency <19 or TICS-M word recall ≤10/20, then subjects were eligible. Alternatively, if TICS-M was <17 but category fluency was ≥19 or word recall was ≥10/20, then subjects were also eligible. Other measures to confirm the MCI diagnosis collected at the first visit were a Mini-mental state examination (MMSE) [30] score of >24/30 and no evidence of dementia. Further details about the protocol are in Methods S1 and in Protocol S1.

Exclusion criteria included: a diagnosis of dementia or being treated with anti-dementia drugs; active cancer; major stroke within past 3 months; treatment with methotrexate, anti-cancer or anti-epileptic drugs, or taking folic acid >300 µg/d, pyridoxine >3 mg/d or vitamin B12 >1.5 µg/d by mouth or any dose by injection. Those taking B vitamins below these doses were allowed to continue during the trial.

Subjects with MCI who also fulfilled entry criteria and gave written consent, were randomised to either a treatment group or a placebo group. Centralised telephone randomization by independent statisticians was used with full allocation concealment and minimization for age, gender, baseline TICS-M score and consent for MRI. Tablet containers were labelled only with the name of the trial and the allocated concealment number and the participants, care partners, and all staff directly involved in the trial were blinded to interventions during the period of the trial. The treatment group received oral TrioBe Plus® (Meda AB/Recip AB, Box 906, Pipers väg 2A, SE-170 09 Solna, Sweden) containing 0.8 mg folic acid, 0.5 mg cyanocobalamin and 20 mg pyridoxine HCl, or a placebo tablet (see Methods S1). The treatment period was 2 years. Each participant received their study medication at first visit and by post at 6-monthly intervals. For those who consented to the MRI scans, the tablets were dispensed on the day of the first scan. At the second visit, or the second MRI scan, participants handed back the tablet bottles. Blood sampling and biochemical assays are described in Methods S1.

### MRI scans

Volumetric cranial MRI scans both at baseline and after 2 years were carried out on a 1.5T MRI system (Sonata; Siemens Medical Solutions, Erlangen, Germany) at the Oxford Centre for Clinical Magnetic Resonance Research. The protocol was T1-weighted acquisition, gradient echo (FLASH-Fast Low angle shot) 3D acquisition with 1 mm isotropic voxels Flip angle 19 degrees TR = 12 ms TE = 5.65 ms; 208 slices per slab with 1 slab acquired in coronal orientation, 1 average. This was repeated three times and averaged after acquisition and cross-repeat alignment.

A fully automated, quantitative method, SIENA, was used to derive the rate of whole brain atrophy per year. SIENA is accurate with high robustness [31]. The rate of change is estimated from two MR images taken at different time points. SIENA automatically segments brain from non-brain in each image, and estimates the external surface of the skull in each image. The two brain images are registered, while using the skull images to constrain scaling and skew; this corrects for changes in imaging geometry over time. Brain surface points (including ventricle surfaces) are found using the registered brain images to sub-voxel accuracy, and the surface motion estimated on the basis of these points. The mean perpendicular edge motion across the entire brain surface produces a change image that can be converted into estimates of rate of atrophy reflecting changes in both grey and white matter [31]. In this study, we have used the rate of atrophy per year based on measurements two years apart.

A cross-sectional method (SIENAX) was used to estimate normalized brain volume from a single image, using the skull to normalize spatially, with respect to a standard image [31]. A participant's normalized brain volume at baseline was used as a covariate in some of the analyses.

### Statistical Analysis

Power estimates for the trial were based on existing data using the same MRI procedure and SIENA in 49 elderly with MCI from OPTIMA where the mean (SD) rate of shrinkage was 0.74 (0.27)% per year. Similar values were reported by Jack et al. [5]. To detect a 20% reduction in rate, we needed 70 subjects per group for 90% power, or 50 subjects per group for 80% power at alpha = 0.05 (two tailed). On the basis of a drop-out rate or failed MRI of ∼20%, we aimed for a sample size of 90 in each arm at the start of the study. This study was not powered for assessment of cognitive effects (to be reported separately).

Variables were treated as normally distributed in the population unless otherwise found by standard tests, in which case they were log transformed. Those values that were log transformed are shown in Table S1.

The efficacy analyses were performed on the basis of the intention-to-treat principle, and included all randomly assigned subjects who received at least one dose of the assigned study medication. The main outcome measure was to determine whether the rate of atrophy of the whole brain per year over the trial period differed between the treatment groups, using the SIENA method. Since the requirement was that the subjects had both a baseline and a follow-up MRI, we conducted an intention-to-treat analysis for the main outcome in the subgroup that completed both MRI scans (n = 168). Plasma vitamin response was reported from the same group. Serious adverse events were evaluated in the total intention-to-treat group (n = 266/271).

We also analyzed the rate of atrophy according to biological compliance, defined by changes in plasma vitamin concentrations over two years, using the following cut-off values for the two groups. In the active treatment group compliant subjects were defined as those who responded with increases from baseline to follow-up in plasma folate of >10 nmol/L and in vitamin B12 of >150 pmol/L. In the placebo group compliant subjects were defined as those who showed increases of ≤10 nmol/L in folate and ≤150 pmol/L in vitamin B12. The other subjects were classified as biologically non-compliant subjects.

Age was considered a confounding variable for the primary endpoint [3]. A variety of other covariates that might be associated with rate of brain atrophy [5], [32], [33], [34], [35], [36] or with B vitamin status [37] were identified before the study was analysed. The assessment of the influence of covariates included a univariate procedure (unpaired t test or Pearson's correlations), followed by age-adjusted analyses. If any variable was significant in the age-adjusted analyses at *P*<0.10, it was included in subsequent analyses. Differences between intervention groups were tested using the Chi square test for categorical variables and the *t* test or analysis of variance for continuous variables.

Pre-specified secondary outcomes included cognitive and depression scores (to be reported separately), serious adverse events, withdrawals, compliance and the changes in biochemical markers. Pre-specified subgroup analyses using ANOVA (categorical variables) or linear regression (continuous variables) included the influence of baseline vitamin markers on treatment effect, the rate of atrophy in the two groups after evaluation of biochemical compliance, association between change in biochemical markers (independent of treatment code) and rate of atrophy, and rate of atrophy in relevant subgroups . Reported *P* values are 2-sided and unadjusted for multiple comparisons; *P* <0.05 was regarded as statistically significant. SPSS for Macintosh (16^th^ ed.) or Windows (17^th^ ed.), (SPSS Inc, Chicago, IL; USA) was used for the statistical analyses.

## Results

### Participants

**Figure 1:**
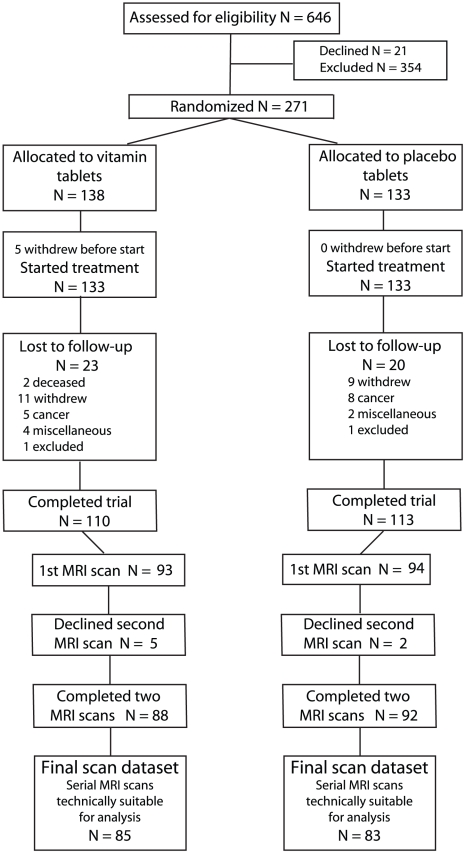
Participant Flow in the trial.

The flow of participants through the study is shown in Fig. 1. From a total of 646 participants assessed through the initial telephone interview, 292 fulfilled the entry criteria. A total of 271 subjects was randomized, but five subjects did not start treatment and are excluded from the intention-to-treat analyses. The numbers lost to follow-up were similar in both groups, with 110 and 113 completing the 24 month trial in the active group and placebo group, respectively. The primary analysis included only those subjects where we had technically good MRI scans at baseline and at follow-up, i.e., 85 in the active group and 83 in the placebo. The baseline characteristics in the groups were similar (Tables 1 and 2). The mean (SD) period between MRI scans was 24.3 (0.7) months.

**Table 1:** Baseline characteristics of the participants completing the MRI protocol.

Characteristics	Placebo group (n = 83)		Active treatment group^a^ (n = 85)	
	Mean or n	SD or %	Mean or n	SD or %
Age, years	76.2	4.5	77.0	5.2
Women, n (%)	52	62.7	50	58.8
Years of education	14.8	3.5	14.3	3.6
Body Mass Index, kg/m^2^ b	26.6	4.2	25.3	3.4
Systolic blood pressure, mmHg	147	19	148	25
Diastolic blood pressure, mmHg	80	11	80	11
TICS-M score	24.8	2.7	24.9	2.8
MMSE score	28.3	1.5	28.3	1.8
Initial brain volume, mL	1376	71	1387	86
Depression score (GDS) c	7.5	5.2	5.6	4.0
Ever-smoker, n (%)	43	51.8	38	44.7
No ankle vibration sense, n (%)	50	60.2	55	64.7
Hemoglobin, g/L	138	12	138	13
MCV, fL	93.0	4.3	92.3	4.4
Creatinine, µmol/L	97	17	96	18
*APOE* ε4 positive, n (%)	29	34.9	22	25.9
*MTHFR* 677C>T allele frequency (%)		34.9		34.7
*TCN2* 776C>G allele frequency (%)		30.1		37.1
Use of B vitamins at baseline, n (%)	17	20.5	14	16.5
Use of fish-oils, omega-3, n (%)	31	37.3	36	42.4
Diabetes any time, n (%)	10	12.05	4	4.7
Use of CVD drugs baseline, n (%)	36	43.4	42	49.4
Use of centrally acting drugs, n (%)	20	24.1	23	27.1
Use of aspirin baseline (%)	28	33.7	26	30.6
Other NSAIDs baseline	12	14.5	18	21.2
Stroke, TIA, MRI infarct at baseline	15	18.1	13	15.3
History of MI baseline	6	7.3	6	7.1
Alcohol consumption (units/week)	7.2 d	8.6	8.2	9.3

Abbreviations: *APOE*, gene for apolipoprotein E; CVD, cardiovascular disease; GDS, Geriatric Depression Scale; MCV, mean red cell volume; MI, myocardial infarct; MMSE, mini-mental state examination; *MTHFR*, gene for methylenetetrahydrofolate reductase; NSAID, non-steroidal anti-inflammatory drug; TIA, transient ischemic attack; TICS-M, telephone interview of cognitive status, modified. Except where indicated by superscript letters, none of the factors differed between the placebo and active treatment groups.

a Active treatment group received daily supplements of folic acid (0.8 mg), vitamin B_12_ (0.5 mg) and vitamin B_6_ (20 mg) for 24 months.

b *P* = 0.022;

c *P* = 0.009;

d Excluding one high outlier.

**Table 2:** Folate and vitamin B12 markers in plasma before and after 2 years of intervention in the MRI subgroup.

			Placebo group		Active treatment group^a^			
		N	Geometric mean	95% C.I.	N	Geometric mean	95% C.I.	*P value* b
**tHcy**	Before	83	11.27	(10.58–12.00)	85	11.25	(10.58–11.97)	*0.974*
**(µmol/L)**	After	83	12.14	(11.40–12.93)	84	8.72	(8.29–9.17)	***<0.001***
*P value* c			***<0.001***			***<0.001***		
**Folate (nmol/L)**	Before	83	24.2	(21.4–27.5)	85	22.4	(19.4–25.9)	*0.428*
	After	83	24.9	(21.4–29.1)	84	82.1	(74.6–90.4)	***<0.001***
*P value* c			*0.695*			***<0.001***		
**Vitamin B_12_ (pmol/L)**	Before	83	333	(310–357)	85	330	(303–360)	*0.891*
	After	83	366	(335–400)	84	672	(626–722)	***<0.001***
*P value* c			***0.018***			***<0.001***		
**HoloTC (pmol/L)**	Before	83	68	(61–76)	85	63	(55–72)	*0.406*
	After	83	73	(65–82)	84	182	(162–204)	***<0.001***
*P value* c			*0.116*			***<0.001***		
**TC saturation (%)**	Before	83	7.35	(6.54–8.25)	85	6.65	(5.72–7.73)	*0.306*
	After	83	7.17	(6.26–8.21)	84	20.42	(18.21–22.90)	***<0.001***
*P value* c			*0.648*			***<0.001***		
**Cystathionine**	Before	83	0.303	(0.273–0.337)	85	0.265	(0.237–0.295)	*0.082*
**(µmol/L)**	After	83	0.350	(0.311–0.395)	84	0.215	(0.196–0.235)	***<0.001***
*P value* c			***0.002***			***<0.001***		

Abbreviations: HoloTC, holotranscobalamin; TC saturation, ratio of holoTC to total TC; tHcy, plasma total homocysteine.

a Active treatment group received daily supplements of folic acid (0.8 mg), vitamin B_12_ (0.5 mg) and vitamin B_6_ (20 mg) for 24 months.

b Student's t-test for unpaired samples,

c Student's t-test for paired samples.

### Adherence, and biological vitamin response

Adherence, assessed by counting returned tablets, was overall good in both groups: More than 78% of participants used at least 75% of their medication. Adherence was also assessed by measuring plasma vitamins and related compounds (Table 2). In the active treatment group, geometric mean (95% CI) of plasma folate increased by nearly 270% and plasma vitamin B12 doubled. In contrast, the corresponding changes for the placebo group were modest increases of 3% and 10%, respectively. Plasma tHcy decreased by 22.5% in the active group, but increased by 7.7% in the placebo group. Using criteria for biological compliance based on changes in folate or vitamin B12, as defined in Methods, we found that 17 out of 83 (20.5%) of the placebo group had taken supplementary folic acid or vitamin B12. In the active treatment group, 14 out of 84 subjects (16.7%) with blood samples available did not take, or did not absorb, the vitamins, at least in the period prior to the second blood sampling (at 24 months). Thus, altogether 136 subjects are defined as biologically compliant out of the 167 in the MRI sub-study where plasma samples were available.

### Factors associated with rate of atrophy in placebo group

Several factors that might influence the rate of atrophy, tHcy or B vitamin status and/or cognition were pre-specified and tested for their effects; these are shown in Tables S1 and S2. Age was strongly associated with rate of brain atrophy (r = 0.32, *P*<0.01) and so all subsequent analyses were adjusted for age. Neither sex, smoking, BMI, alcohol consumption, *APOE*4 status nor *MTHFR* 677C>T polymorphism was associated with the rate of atrophy (*P*>0.1 for all, adjusted for age). For the continuous variables, rate of atrophy was significantly associated with baseline log tHcy (partial r = 0.41, *P*<0.001) and with plasma creatinine (partial r = 0.22, *P* = 0.049). Borderline associations were observed for diastolic blood pressure (partial r = **−**0.21, *P* = 0.054), and for initial brain volume (partial r = **−**0.19, *P* = 0.092). The latter four variables were included in subsequent adjusted analyses.

### Main outcome: rate of atrophy

Treatment with B vitamins for 24 months significantly slowed the rate of brain atrophy. After adjustment for age, the rate of brain atrophy per year was 29.6% less in the active treatment group (0.76% [95% CI, 0.63–0.90]) compared to the placebo group (1.08% [0.94–1.22], *P* = 0.001). Additional adjustment for the above-mentioned variables marginally changed the decrease to 27.1% (respective rates of atrophy: 0.78% [0.64–0.91] and 1.07% [0.94–1.21], *P* = 0.003). If we confined the analysis to the biologically compliant subjects (n = 136), the effect of treatment was slightly greater with a reduction in atrophy rate of 31.1% in the active treatment group (rate of atrophy: 0.73% [0.57–0.88]) compared to the placebo group (1.06% [0.90–1.22], *P* = 0.004 after multi-adjusted analysis). There was no effect of treatment on the 31 subjects who were categorised as biologically non-compliant (*P* = 1.00), probably because they started with a lower tHcy at baseline and, in those receiving B vitamins, the tHcy reduction was a modest 13%. Furthermore, the number of subjects is too small to detect an effect.

### Secondary outcomes

**Figure 2:**
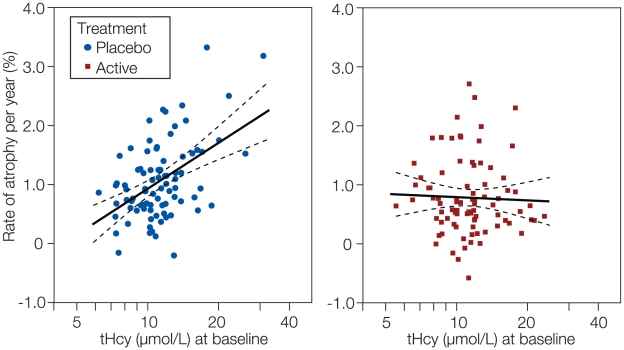
Atrophy rate by baseline homocysteine. Linear regression lines with 95% mean prediction intervals. R^2^ for placebo group (n = 83) was 0.242 (*P*<0.001); for the treatment group (n = 85) R^2^ was 0.001 (*P* = 0.74). The x-axis is a logarithmic scale labelled with linear values.

As stated above, the subjects responded to the treatment as expected in terms of plasma vitamin response (Table 2). Furthermore, we found a significant interaction between baseline tHcy level and treatment effect (log tHcy x treatment, *P* = 0.001). In the placebo group, tHcy at baseline showed a striking positive relationship to the rate of atrophy (R^2^ = 0.24), whereas this association was absent in the active group (Fig. 2). Neither baseline folate nor the vitamin B12 markers showed such a relation.

**Figure 3:**
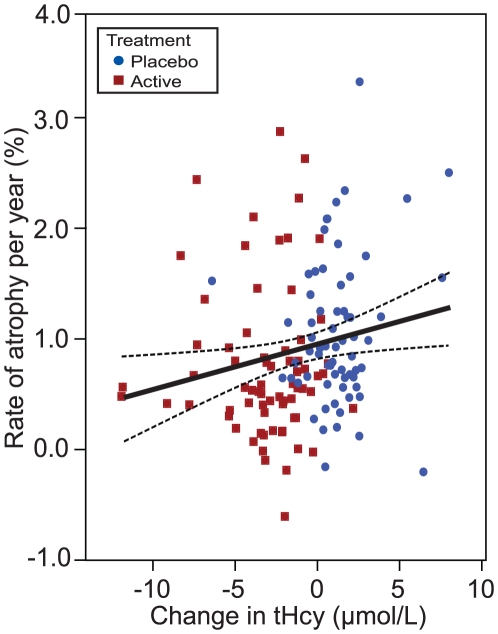
Atrophy rate by change in plasma total homocysteine over a two year period. Subjects in this analysis were a subset (66 in placebo; 70 in the active treatment) who showed biochemical evidence of good compliance (see Table 3). Linear regression with 95% mean prediction intervals, adjusted for age at baseline; partial r = 0.22, *P* = 0.011. The x-axis is a logarithmic scale labelled with linear values.

**Figure 4:**
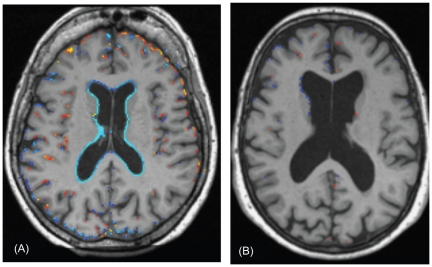
Selected subtraction MRI scans. The images are from the baseline scan with colour superimposed to show the brain tissue change over the following two years. Colours show expansion (red/yellow) or contraction (blue/light blue) of the brain of 0.3 to 1.0 mm, with the lightest colour indicating the biggest change. (A) Subtraction image of female participant in the placebo group, age 79 years, with baseline tHcy of 22 µmol/L, whose tHcy concentration increased by 8 µmol/L over two years. Atrophy rate was 2.50% per year. Atrophy most strongly appears here as enlargement of the ventricles. (B) Subtraction image of female participant in active treatment group, age 72 years, with baseline tHcy of 24 µmol/L at baseline, whose tHcy concentration decreased by 12 µmol/L over two years. Atrophy rate 0.46% per year. There is no clear visible pattern of atrophy.

We also examined atrophy in relation to the change in tHcy, folate and vitamin B12 markers from baseline to follow-up (Table 3). Rate of atrophy was significantly associated with the change in tHcy, and inversely with change in holoTC and TC saturation. Furthermore, when we confined the analyses to the biologically compliant subjects, the effects became stronger and change in folate and vitamin B12 also became significant. There was no association with change in cystathionine levels (a marker of vitamin B6 status). Thus, the greater the improvement in folate or vitamin B12 status, the slower was the rate of atrophy. Conversely, those subjects whose folate or vitamin B12 status declined were at increased risk of atrophy, illustrated in Fig. 3, using tHcy as a marker. A striking example of the effect of changes in tHcy concentrations on brain atrophy over two years is shown in Fig. 4, which illustrates subtraction cranial MRI scans from a participant in the placebo group whose tHcy concentration increased (Fig. 4A), and from a member of the active treatment group whose tHcy concentration decreased (Fig. 4B). Both subjects started with similar raised tHcy concentrations but the participant taking placebo showed a further increase in tHcy over the two years, while the participant taking active treatment showed a marked fall in tHcy over this period. The rate of atrophy was more than 5-times slower in the participant taking B vitamins than in the subject taking placebo.

**Table 3:** Associations of rate of atrophy with changes in plasma biochemical markers upon treatment^a^.

Change in	As randomised^b^		Compliant subjects^c^	
Marker	Partial r^d^		Partial r^d^	
	(n = 166)	*P*	(n = 134)	*P*
tHcy	0.19	**0.017**	0.25	**0.004**
Folate	**−**0.13	0.096	**−**0.29	**0.001**
Vitamin B_12_	**−**0.05	0.516	**−**0.27	**0.002**
HoloTC	**−**0.20	**0.011**	**−**0.25	**0.004**
TC saturation	**−**0.22	**0.004**	**−**0.25	**0.005**
Cystathionine	0.06	0.472	0.03	0.708

Abbreviations: HoloTC, holotranscobalamin; TC saturation, ratio of holoTC to total TC; tHcy, plasma total homocysteine.

a Active treatment group received daily supplements of folic acid (0.8 mg), vitamin B_12_ (0.5 mg) and vitamin B_6_ (20 mg) for 24 months.

b All subjects in the MRI subgroup that started treatment and with baseline and follow-up blood sampling.

c The actively treated subjects where classed as compliant if they had an increase from baseline to follow-up in plasma folate of >10 nmol/L and in vitamin B12 of >150 pmol/L; the placebo subjects were classed as compliant if the increase from baseline to follow-up was ≤10 nmol/L in plasma folate and ≤150 pmol/L in plasma vitamin B12. ^d^Adjusted for age, baseline diastolic blood pressure, baseline creatinine, initial brain volume and log baseline tHcy.

The effects in various subgroups are shown in Table S2. There were no significant interactions between treatment and the following variables: age, sex, category of MCI, normalized initial brain volume, hypertension, use of non-aspirin NSAIDs, smoking, creatinine, *APOE*4 and *MTHFR* 677C>T. Diabetes was associated with increased rate of atrophy (*P* = 0.014) and this effect was apparently not influenced by treatment, although the interaction was not significant, possibly because of the small number of subjects (n = 4). In line with the interaction with baseline tHcy described above, in participants with baseline tHcy below the median, the active treatment was associated with 11.2% slower rate of atrophy, whereas those with baseline tHcy above median showed a 43.0% reduction in atrophy (*P*_interaction_ = 0.019). When tHcy was further divided into quartiles, there was no effect of treatment in those in the lowest quartile (tHcy ≤9.5 µmol/L), whereas there was a 53.3% reduction in rate of atrophy in those in the 4^th^ quartile of tHcy (>13.0 µmol/L) treated with B vitamins vs. placebo (*P*_treatment_ = 0.001; *P*_tHcy_ = 0.139; *P*_interaction_ = 0.023). An interaction between treatment category and a history of stroke or TIA at baseline was found: those in the placebo group with a previous event had an atrophy rate per year of 1.76%[1.31–2.21] compared with 1.01% [0.86–1.15] for those without an event. Those in the active treatment group had rates of 0.74% [0.35–1.14] and 0.77% [0.63–0.91] (*P*_treatment_<0.001; *P*_stroke_ = 0.028; *P*_interaction_ = 0.014), respectively. Thus, the increased rate in the stroke subjects was slowed down by the vitamin treatment. This interaction with stroke was no longer significant (*P* = 0.098) when subjects with silent infarcts seen on MRI were included, although there was still a significant effect of stroke overall on atrophy rate (*P* = 0.025). Regular use of aspirin showed a tendency to interact with treatment (*P*_treatment_ = 0.021; *P*_aspirin_ = 0.19; *P*_interaction_ = 0.052); in those taking aspirin the treatment appeared less effective. A subset of participants reported taking multivitamin supplements containing B vitamins prior to the trial (Table 1) and in these there was a significant interaction with treatment (*P* = 0.034) such that active treatment was no longer effective. This lack of effect may be related to their low tHcy (geometric mean 9.9 [9.0–10.9] µmol/L), high folate (38.1 [30.9–46.9] nmol/L) and vitamin B12 (388 [343–441] pmol/L) already at baseline. This baseline concentration of tHcy is close to that described above (9.5 µmol/L) where we found no effect of treatment.

Although the study was not powered to detect an effect of treatment on cognition (findings to be reported separately), in a *post hoc* analysis, we noted that final cognitive test scores were correlated to the rate of atrophy. Multiple linear regression showed that the main factors that significantly determined the MMSE score at the end of the study were baseline MMSE score (partial r = 0.42, *P*<0.001), rate of brain atrophy (partial r = **−**0.36, *P*<0.001) and age (partial r = **−**0.20, *P* = 0.01); the adjusted R^2^ was 0.33. The same factors determined the final TICS-M score: baseline TICS-M (partial r = 0.39, *P*<0.001), atrophy rate (partial r = **−**0.36, *P*<0.001), and age (partial r = **−**0.27, *P*<0.001); the adjusted R^2^ was 0.39. Thus, brain atrophy rate appears to be a major determinant of cognitive decline in this population.

### Safety outcomes in the whole cohort

The overall B vitamin status was very good in the whole cohort of 266 participants. Only 7 participants (2.6%) had plasma folate concentrations of <7 nmol/L and 6 (2.3%) had vitamin B_12_ concentrations of <150 pmol/L at baseline. Since the vitamin analyses were done after the trial ended, these subjects, although classified as vitamin deficient, were not treated medically unless diagnosed by their GP.

Altogether 48 subjects were lost to follow-up in the whole trial, 28 (five immediately before starting taking tablets) in the active group and 20 in the placebo group. Reasons for withdrawal are shown in Table S3. There were no significant safety issues and no significant differences in adverse events, except that there were fewer subjects in the active treatment group who showed a loss of vibration sense (Table S3). The time to dropout was shorter in the active group, even after excluding the immediate dropouts.

## Discussion

B vitamin treatment led to a difference in final tHcy concentration of 31.7% compared with the placebo, and was accompanied by a reduction in the rate of brain atrophy of almost 30%. No safety issues were found, so it can be concluded that high doses of B vitamins can be used to reduce the rate of atrophy of the brain in elderly people with MCI.

In agreement with the prior hypothesis, the treatment effect was greatest in those with the highest baseline level of tHcy, with a reduction in atrophy rate of 53% in those in the top quartile of tHcy (>13.0 µmol/L). Notably, there was no effect of treatment on atrophy in those in the bottom quartile (≤9.5 µmol/L). The observation in the placebo group that the rate of atrophy was related to the baseline concentration of tHcy is consistent with, and may explain, a number of cross-sectional studies reporting that regional brain atrophy is related to tHcy [17], [18], [19], [20], [21]. The results also fit with our earlier finding that raised tHcy predicts the rate of shrinkage of the medial temporal lobe in patients with Alzheimer's disease [12]. In contrast, in the group on active treatment there was no relationship between baseline tHcy and the rate of atrophy. It is tempting to suggest that this finding is consistent with the view that raised homocysteine is a direct cause of the atrophy. However, it does not exclude that tHcy is only a marker for low-normal levels of the vitamins, which are themselves the causal factors.

Our previous observational study on another elderly cohort in OPTIMA showed that low-normal baseline levels of vitamins B_12_ were associated with a more rapid rate of atrophy, whereas folate levels were unrelated [36]. In the present study, we found that an increase in either vitamin B_12_ status or in folate status was associated with a reduced rate of atrophy. Thus, from the present data we cannot decide which of these two vitamins is the most important. The lack of association of atrophy with the change in cystathionine levels, a marker of vitamin B_6_ status [38], may indicate that vitamin B_6_ is less important as a determinant. It is noteworthy that a cross-sectional study found that supplementary intake of vitamin B_6_ or of vitamin B_12_, but not of folate, was associated with greater regional gray matter volumes in an elderly cohort [39].

### Possible therapeutic implications

We have shown that treatment for two years with B vitamins markedly slows the accelerated rate of atrophy in people with MCI. This study was carried out in the UK, where voluntary fortification of foods with folic acid is permitted but where there is no mandatory fortification. The effect of treatment was dependent on baseline tHcy, with those in the upper three quartiles, i.e. >9.5 µmol/L, showing a significant slowing of atrophy upon treatment compared with those in the lowest quartile. In the USA, which has mandatory fortification, 13.6% of those ≥60 years-old had tHcy concentrations >13 µmol/L in 2003-4 [40], a level at which we found a >50% reduction in the rate of atrophy upon treatment with high doses of B vitamins. The median tHcy concentration in those ≥60 years-old in the USA is 10.1 µmol/L, suggesting that a substantial proportion of those with MCI could benefit from the intervention. The prevalence of MCI is between 14% and 18% in those over 70 years-old [24], [41], which means that about 5 million people in the USA and 14 million in greater Europe suffer from this condition. Since approximately half of those with MCI convert to Alzheimer's disease or to another form of dementia within 5 years [42], there is an urgent need to identify treatments that will slow down or prevent the conversion [43]. The outcome of the VITACOG trial indicates that treatment with homocysteine-lowering B vitamins might be one approach to meeting this challenge,

This study was designed to detect an effect of treatment on the rate of atrophy and was not powered to detect effects of treatment on cognitive test scores. Nonetheless, we consider that the findings are relevant to cognitive decline in people with MCI. First, in studies over longer periods (up to 5 years) it has been found that the rate of whole brain atrophy in MCI is correlated with cognitive decline in several tests, including the MMSE [5]. Second, when we looked for significant predictors of the final cognitive test score, the rate of atrophy was one of the three main factors determining the final MMSE and TICS-M scores. Third, two other randomised controlled trials of homocysteine-lowering treatments have shown effects on cognition: a trial in which normal participants with baseline tHcy levels >13 µmol/L were treated with folic acid (0.8 mg/d) for three years showed a beneficial effect on several cognitive tests [44]. An 18-month trial of high doses of the same three vitamins used here showed a slowing of cognitive decline in patients with mild Alzheimer's disease, although not in patients with moderate Alzheimer's disease [45]. The latter result indicates that homocysteine-lowering may have to be targeted at those with early stages of Alzheimer's disease. Since the rate of brain atrophy is more rapid in subjects with MCI who convert to Alzheimer's disease [5], it is possible that high doses of folic acid, vitamins B_6_ and B_12_ might slow the conversion from MCI to Alzheimer's disease. Clinical trials to test this hypothesis are warranted.

### Strengths and limitations

A particular strength of the study is that we used a highly sensitive and accurate tool for assessment of brain atrophy, i.e., MRI and the SIENA protocol. Notably, the SIENA analysis was carried out on MRI data derived from the average of three T1 volumetric scans at each time point, so providing a high degree of accuracy. Another strength was the measurement of vitamins and their markers, which made it possible to show that the baseline tHcy concentration is a key determinant of the rate of atrophy and of the response to treatment. In our study, brain atrophy was among the strongest determinants of MMSE and TICS-M at the end of the study, supporting the view that assessment of atrophy rate may be a useful tool in cognitive studies. The study has, however, some limitations. First, we used combination of the three B vitamins, so we cannot identify whether they are all required or if one is more important. Second, this trial was powered to detect change in rate of atrophy, not cognition; even so we observed a strong association between atrophy rate and cognition. A subsequent report will describe the cognitive results in more detail. Finally, although some of the findings in subgroup analyses appear quite striking, for example in the stroke or diabetes groups, or those taking aspirin, these results should be interpreted with caution because of the small sample size and lack of biological explanation for the interaction. The importance of these findings will be in relation to future trials. There is every reason to include those who have had a previous stroke or subjects with high creatinine. We obviously need more data on those using aspirin. In subjects already taking oral supplements of B vitamins, or with good plasma tHcy and B vitamin status, the effect may be limited, but it should be investigated whether subgroups could benefit from even higher intakes, as appeared to be the case for those with mild AD and with lower levels of tHcy than in our cohort [45].

### Conclusions

We show that a simple and safe treatment that targets homocysteine can slow down the accelerated rate of brain atrophy found in mild cognitive impairment. Although this is relatively small trial, it has provided useful data for planning future studies, whether those will focus on brain atrophy, cognitive decline, or age-related conditions where brain atrophy will be included as part of the assessment.

## Supporting Information

Methods S1 Text file with additional details of methods.
(0.02 MB PDF)

Table S1 (0.08 MB PDF)

Table S2 (0.07 MB PDF)

Table S3 (0.05 MB PDF)

Protocol S1 VITACOG Trial protocol
(0.13 MB PDF)

Checklist S1 CONSORT Checklist
(0.05 MB PDF)
