# Supplementary material for: Homocysteine-Lowering by B Vitamins Slows the Rate of Accelerated Brain Atrophy in Mild Cognitive Impairment: A Randomized Controlled Trial
Source: PLoS One. 2010 Sep 8;5(9):e12244. doi: 10.1371/journal.pone.0012244 (PMC2935890; doi:10.1371/journal.pone.0012244)
Supplement: Methods S1 — Text file with additional details of methods. (0.02 MB PDF) [file pone.0012244.s001.pdf]

Supplementary Methods

Further study protocol details

Via a telephone interview, respondents completed a health screening questionnaire relating to inclusion and exclusion criteria, and completed the TICS-M and a category fluency test (CERAD) [1,2]. Eligible participants were asked if they would agree to have two cranial MRI scans, one at the start and one two years later at the end of treatment, but it was emphasized that the scans were voluntary. Although the main outcome of the trial was atrophy rate, we allowed subjects that could not or did not wish to participate in the MRI part of the study to enter the trial in a separate arm: the intention was to evaluate recruiting strategies for such trials (not reported here) for subjects with MCI.

At the initial clinic visit, participants provided blood and urine samples and underwent a variety of cognitive tests (to be described separately), including the MMSE [3], and they completed the Geriatric Depression Scale (GDS) questionnaire [4]. Participants also had a simple test of their vibration sense in the ankle with a tuning fork. Participants were contacted by telephone at the following times after starting treatment: 3, 6, 12, 15 and 18 months to check compliance, ask about adverse events and to carry out a verbal memory test. After 24 months, participants returned to the clinic for examination, blood tests described in Table 1; the cognitive test battery was repeated as well. A final telephone assessment of adverse events and cognition was done at 27 months.

Placebo tablet

The placebo tablet was identical to the vitamin tablet (TrioBe Plus®) except for omission of the vitamins and the addition of iron oxide and ferrous sulphate (0.0055%) to give a colour to match the vitamin tablet.

Blood sampling and assays

At baseline and after 2 y, non-fasting blood samples were collected by venipuncture. Plasma tHcy, folate, cobalamin, holoTC, and total TC were determined as previously described [5]. TC saturation was calculated as a ratio of holoTC to total TC. Plasma cystathionine was determined by liquid chromatography tandem mass spectrometry [6].

Genomic DNA was extracted from blood using the Wizard DNA Purification Kit (Promega, Southampton, UK). The *MTHFR* 677C>T polymorphism (NCBI Entrez Gene 4524) and the *TCN2* 776C>G polymorphism (NCBI Entrez Gene 6948) were genotyped using the Amplifluor SNP Genotyping System (Chemicon, Watford, UK) [7], while *APOE* genotypes (NCBI Entrez Gene: 348) was determined using a one-stage PCR method [8].

REFERENCES

1. De Jager CA, Budge MM, Clarke R (2003) Utility of TICS-m for the assessment of cognitive function in older adults. Int J Geriatr Psychiatry 18: 318-324.
2. Morris JC, Heyman A, Mohs RC, Hughes JP, Vanbelle G, et al. (1989) The Consortium to Establish a Registry for Alzheimers Disease (CERAD). Part 1.Clinical and Neuropsychological Assessment of Alzheimers Disease. Neurology 39: 1159-1165.
3. Folstein MF, Folstein SE, McHugh PR (1975) Mini-mental state: a practical method of grading the cognitive state of patients for the clinician. J Psychiatr Res 12: 189-198.
4. Yesavage JA, Brink TL, Rose TL, Lum O, Huang V, et al. (1982) Development and validation of a geriatric depression screening scale: a preliminary report. J Psychiatr Res 17: 37-49.
5. Vogiatzoglou A, Refsum H, Johnston C, Smith SM, Bradley KM, et al. (2008) Vitamin B12 status and rate of brain volume loss in community-dwelling elderly. Neurology 71: 826-832.
6. Antoniades C, Shirodaria C, Leeson P, Baarholm OA, Van-Assche T, et al. (2009) MTHFR 677 C>T polymorphism reveals functional importance for 5-methyltetrahydrofolate, not homocysteine, in regulation of vascular redox state and endothelial function in human atherosclerosis. Circulation 119: 2507-2515.
7. Warden DR, Refsum H (2005) Detection of single-nucleotide polymorphisms by PCR with universal energy transfer-labeled primers: application to folate- and cobalamin-related genes. Clin Chem 51: 1713-1716.
8. Wenham PR, Price WH, Blandell G (1991) Apolipoprotein E genotyping by one-stage PCR. Lancet 337: 1158-1159.
