# Supplementary material for: Homocysteine-Lowering by B Vitamins Slows the Rate of Accelerated Brain Atrophy in Mild Cognitive Impairment: A Randomized Controlled Trial
Source: PLoS One. 2010 Sep 8;5(9):e12244. doi: 10.1371/journal.pone.0012244 (PMC2935890; doi:10.1371/journal.pone.0012244)
Supplement: Table S1 — (0.08 MB PDF) [file pone.0012244.s002.pdf]

Supplementary Table S1. Pearson correlations with the rate of atrophy (% per year) in the placebo group (N=83)

	**Unadjusted Correlation**	***P***	**Age-adjusted correlation**	***P***
Age at first visit	**0.317**	**0.004**		
Initial brain volume	**−0.319**	**0.003**	-0.187	0.092
Total schooling	0.011	0.923	0.030	0.787
Body mass index at baseline	0.056	0.615	0.064	0.565
Diastolic blood pressure at baseline	**−0.310**	**0.004**	**−0.213**	**0.054**
Systolic blood pressure at baseline	−0.101	0.362	−0.084	0.455
Creatinine at baseline	**0.274**	**0.012**	**0.218**	**0.049**
Depression score (GDS) at baseline	0.156	0.158	0.148	0.184
Cystathionine at baseline	0.101	0.363	0.102	0.362
Log folate at baseline	−0.062	0.58	−0.092	0.411
Log vitamin B_{12} baseline	−0.052	0.644	−0.002	0.988
Log holoTC at baseline	−0.183	0.098	−0.125	0.264
Log TC saturation at baseline	−0.268	**0.014**	−0.180	0.105
Log tHcy at baseline	**0.492**	**<0.001**	**0.407**	**<0.001**

Abbreviations: GDS, Geriatric Depression Scale; HoloTC, holotranscobalamin; TC saturation, ratio of holoTC to total TC; tHcy, total homocysteine. Analytes whose values were not normally distributed in the population were log transformed prior to analysis.
