# Supplementary material for: Homocysteine-Lowering by B Vitamins Slows the Rate of Accelerated Brain Atrophy in Mild Cognitive Impairment: A Randomized Controlled Trial
Source: PLoS One. 2010 Sep 8;5(9):e12244. doi: 10.1371/journal.pone.0012244 (PMC2935890; doi:10.1371/journal.pone.0012244)
Supplement: Table S2 — (0.07 MB PDF) [file pone.0012244.s003.pdf]

Supplementary Table S2.
Subgroup analyses: effects of several factors on the rate of atrophy and on the treatment effect

	Placebo				Active treatment[a]			*P* value[b]		
**Factor**	**N**	**Atrophy rate (95% CI) %/y**			**N**	**Atrophy rate (95% CI) %/y**		**Treatment**	**Factor**	**Interaction**
Age≤75.00 years	36	0.95	(0.74-1.17)		39	0.64	(0.43-0.84)	**0.004**	**0.023**	0.824
Age>75.00 years	47	1.16	(0.98-1.35)		46	0.89	(0.70-1.08)			
Male	31	1.04	(0.81-1.26)		35	0.85	(0.64-1.07)	**0.004**	0.707	0.267
Female	52	1.11	(0.94-1.29)		50	0.70	(0.52-0.88)			
Amnestic MCI	51	1.15	(0.97-1.33)		46	0.87	(0.67-1.06)	**0.004**	0.107	0.741
Non-amnestic MCI	26	1.00	(0.75-1.26)		31	0.65	(0.42-0.88)			
Never smoker	40	1.02	(0.82-1.23)		47	0.75	(0.56-0.93)	**0.002**	0.453	0.708
Ever smoker	43	1.14	(0.94-1.33)		38	0.78	(0.58-0.99)			
No aspirin, baseline	55	1.11	(0.94-1.27)		59	0.66	(0.50-0.82)	**0.021**	0.190	**0.052**
Aspirin at baseline	28	1.04	(0.80-1.27)		26	1.00	(0.76-1.24)			
No NSAID at baseline	71	1.08	(0.93-1.23)		67	0.77	(0.61-0.92)	**0.015**	0.960	0.958
NSAID at baseline	12	1.08	(0.72-1.50)		18	0.75	(0.45-1.05)			
No CVD drug, baseline	47	0.97	(0.79-1.16)		43	0.76	(0.57-0.96)	**0.001**	0.234	0.213
CVD drug at baseline	36	1.22	(1.01-1.43)		42	0.76	(0.57-0.96)			
No antihypertensive	56	1.08	(0.91-1.25)		51	0.74	(0.56-0.92)	**0.003**	0.709	0.849
Antihypertensive at baseline	27	1.10	(0.85-1.34)		34	0.80	(0.58-1.02)			
No TIA/stroke at baseline	75	1.01	(0.86-1.15)		75	0.77	(0.63-0.91)	**<0.001**	**0.028**	**0.014**
TIA/stroke at baseline	8	1.76	(1.31-2.21)		10	0.74	(0.35-1.14)			
No stroke, TIA, MRI infarct at baseline	68	0.99	(0.84-1.14)		72	0.75	(0.61-0.90)	**0.001**	**0.025**	0.098
Stroke, TIA or MRI infarct at baseline	15	1.51	(1.18-1.83)		13	0.84	(0.49-1.18)			

**Supplementary Table S2 contd. 2**										
	**Placebo**				**Active treatment[a]**			***P* value[b]**		
**Factor**	**N**	**Atrophy rate (95% CI) %/y**			**N**	**Atrophy rate (95% CI) %/y**		**Treatment**	**Factor**	**Interaction**
CNS drugs at baseline	63	1.03	(0.87-1.18)		62	0.74	(0.58-0.90)	**0.002**	0.17	0.274
No drugs at baseline	20	1.26	(0.98-1.54)		23	0.82	(0.54-1.08)			
No diabetes	73	1.05	(0.90-1.19)		81	0.73	(0.59-0.87)	0.508	**0.014**	0.334
Diabetes anytime	10	1.34	(0.94-1.73)		4	1.40	(0.77-2.02)			
B vitamins, baseline	17	0.86	(0.56-1.17)		14	0.99	(0.65-1.32)	0.246	0.962	**0.034**
No B vitamins	66	1.14	(0.99-1.29)		71	0.72	(0.57-0.87)			
Initial brain < median	43	1.16	(0.97-1.36)		39	0.84	(0.63-1.040	**0.002**	0.157	0.844
Initial brain ≥ median	40	0.99	(0.79-1.20)		46	0.70	(0.52-0.89)			
Without *APOE*4	54	1.07	(0.89-1.24)		63	0.74	(0.58-0.90)	**0.005**	0.523	0.842
With *APOE*4	29	1.11	(0.88-1.35)		22	0.83	(0.56-1.10)			
MTHFR 677CC	33	1.03	(0.81-1.08)		35	0.82	(0.61-1.04)	**0.004**	0.947	0.577
MTHFR 677CT	42	1.10	(0.90-1.05)		41	0.72	(0.52-0.92)			
MTHFR 677TT	8	1.23	(0.78-1.27)		9	0.70	(0.27-1.12)			
TCN2 776CC	21	1.08	(0.80-1.36)		31	0.86	(0.63-1.08)	**0.001**	0.733	0.263
TCN2 776CG	14	1.02	(0.83-1.21)		45	0.75	(0.56-0.94)			
TCN2 776GG	18	1.23	(0.93-1.53)		9	0.51	(0.08-0.93)			
Creatinine ≤ median[c]	39	1.01	(0.81-1.21)		42	0.63	(0.43-0.82)	**0.002**	**0.036**	0.489
Creatinine > median	44	1.15	(0.96-1.34)		42	0.91	(0.71-1.10)			
tHcy ≤ median	44	0.89	(0.70-1.08)		40	0.79	(0.59-0.98)	**0.001**	0.068	**0.019**
tHcy > median	39	1.30	(1.10-1.50)		45	0.74	(0.56-0.93)			
tHcy 1st quartile	20	0.84	(0.57-1.12)		21	0.86	(0.59-1.12)	**0.001**	0.139	**0.023**
2nd quartile	23	0.92	(0.66-1.18)		19	0.71	(0.43-0.99)			
3rd quartile	18	1.05	(0.76-1.34)		24	0.78	(0.53-1.03)			
4th quartile	21	1.52	(1.25-1.79)		21	0.71	(0.44-0.98)			

Supplementary Table S2. contd. 3										
	Placebo				Active treatment[a]			*P* value[b]		
Factor	N	Atrophy rate (95% CI) %/y			N	Atrophy rate (95% CI) %/y		Treatment	Factor	Interaction
Folate < median	39	1.07	(0.87-1.28)		45	0.70	(0.51-0.89)	**0.002**	0.465	0.595
Folate ≥ median	44	1.09	(0.90-1.28)		40	0.83	(0.63-1.03)			
Vitamin B_{12} < median	37	1.07	(0.86-1.28)		47	0.77	(0.58-0.96)	**0.002**	0.976	0.832
Vitamin B_{12} ≥ median	46	1.09	(0.91-1.28)		38	0.75	(0.55-0.96)			
HoloTC < median	37	1.20	(0.99-1.41)		47	0.76	(0.58-0.95)	**0.001**	0.304	0.280
HoloTC ≥ median	46	0.99	(0.80-1.18)		38	0.77	(0.56-0.97)			
TC saturation < median	35	1.22	(1.01-1.44)		45	0.76	(0.57-0.95)	**0.001**	0.261	0.188
TC saturation ≥ median	48	0.98	(0.79-1.16)		40	0.77	(0.57-0.97)			

Abbreviations: *APOE*, gene for apolipoprotein E; CVD, cardiovascular disease; HoloTC, holotranscobalamin; *MTHFR*, gene for methylenetetrahydrofolate reductase; NSAID, non-steroidal anti-inflammatory drug; *TCN2*, gene for transcobalamin-2; tHcy, plasma total homocysteine; TIA, transient ischemic attack

[a]Active treatment group received daily supplements of folic acid (0.8 mg), vitamin B_{12} (0.5 mg) and vitamin B_6 (20 mg) for 24 months. [b]ANOVA was used to examine the effect of each factor on atrophy rate and its possible interaction with the treatment category (two right-hand columns); all analyses were adjusted for age at baseline. [c]Ranked according to sex.
