# Supplementary material for: Homocysteine-Lowering by B Vitamins Slows the Rate of Accelerated Brain Atrophy in Mild Cognitive Impairment: A Randomized Controlled Trial
Source: PLoS One. 2010 Sep 8;5(9):e12244. doi: 10.1371/journal.pone.0012244 (PMC2935890; doi:10.1371/journal.pone.0012244)
Supplement: Table S3 — (0.05 MB PDF) [file pone.0012244.s004.pdf]

Supplementary Table S3. Withdrawals and adverse events

	Placebo (n=133)		Active treatment[a] (n=133)		*P* value[b]
	Mean or n	SD or %	Mean or n	SD or %	
Total withdrawals[c]	20	15.0	23	17.3	
Time to withdrawal (d)	441	179	298	197	**0.017**
Self-withdrawal	9	6.8	11	8.3	
Time to withdrawal (d)	469	115	231	144	**<0.001**
Cancer withdrawal	8	6.0	5	3.8	
Time to withdrawal (d)	370	229	394	186	
Exclusion criterion withdrawal	1		1		
Time to withdrawal (d)	390		433		
Other	2		4		
Time to withdrawal (d)	620	145	231	231	
Change in depression score (GDS)	0.018	3.6	−0.073	3.4	
Loss of vibration sense	13	9.8	3	2.2	**0.019**
Myocardial infarction	1		1		
Stroke	1		3[d]		
Death	0		2[e]		
Time to death (d)			490		
Total adverse events	271		242		

Abbreviation: GDS, Geriatric Depression Scale

[a]Active treatment group received daily supplements of folic acid (0.8 mg), vitamin B_{12} (0.5 mg) and vitamin B_6 (20 mg) for 24 months. [b]Only *P* values < 0.1 are shown. [c]Excluding 5 who withdrew before starting the tablets and 2 who withdrew after the 24 month visit (i.e., after stopping intervention). [d]Includes one of the 2 participants who died. [e]One hemorrhagic stroke; one pulmonary embolism.
