# Supplementary material for: Homocysteine-Lowering by B Vitamins Slows the Rate of Accelerated Brain Atrophy in Mild Cognitive Impairment: A Randomized Controlled Trial
Source: PLoS One. 2010 Sep 8;5(9):e12244. doi: 10.1371/journal.pone.0012244 (PMC2935890; doi:10.1371/journal.pone.0012244)
Supplement: Protocol S1 — VITACOG Trial protocol (0.13 MB PDF) [file pone.0012244.s005.pdf]

Protocol for VITACOG

University of Oxford

Study title:
Homocysteine and B vitamins in cognitive impairment (VITACOG)

Study Code: (REC: 04/Q1604/100)

Eudract Number: 2004-001527-38

Registration: ISRCTN 94410159 (http://www.controlled-trials.com/)

Principal Investigator: Professor Robin Jacoby
Lead investigators: Professors A. David Smith and Helga Refsum
Study Nurse: Pippa Whitbread

Site:
OPTIMA (Oxford Project to Investigate Memory and Aging)
John Radcliffe Hospital
Oxford, OX3 9DU

Sponsoring Institution:
University of Oxford

Ethics Committee: COREC
Local Research Ethics Committee: Oxford A, Approval: COREC 04/Q1604/100

Protocol Version Number 5.2

TABLE OF CONTENTS

Page
1.0 Protocol Synopsis ………………………………………………...4
2.0 **Core Protocol……………………………………………………..6**
2.1 Study Rationale……………………………………………..6
2.2 Aims and Hypotheses……………………………………….6
2.3 Scientific Background………………………………………7
2.4 Trial Design…………………………………………………8
3.0 Detailed Procedures………………………………………… 10
3.1 Screening Procedure…………………………………….10
3.2 Inclusion and Exclusion Criteria………………………….10
3.3 Visit1-MCI Classification and Enrolment………………...10
3.4 First MRI scan visit……………………………………….11
3.5 StudyTreatment…………………………………………11
3.6 3-month Telephone Follow-up……………………………11
3.7 6-month Telephone Follow-up……………………………12
3.8 9-month Telephone Follow-up……………………………12
3.9 12-month Telephone Follow-up…………………………. 12
3.10 15-month Telephone Follow-up………………………12
3.11 18-month Telephone Follow-up………………………12
3.12 21-month Telephone Follow-up………………………12
3.13 Visit 2-24-month Follow-up…………………………..12
3.14 2nd MRI scan visit……………………………………..13
3.15 Post study follow-up by telephone (approx. 27month)..13
3.16 Home Visits……………………………………………13
4.0 **Data Collection ………………………………………………...13**
4.1 Medical…………………………………………………….13
4.2 Biochemical………………………………………………..13
4.3 Genetic……………………………………………………..13
4.4 Cognitive…………………………………………………...13
4.5 Neuropsychological………………………………………...13
4.6 Neuro-imaging ……………………………………………..14
5.0 Study Flow Chart…………………………………………………15
6.0 Safety Assessments………………………………………………..16
6.1 Vitamin Safety……………………………………………..16
6.2 Definitions: (i)Adverse Event……………………………16
(ii) Adverse Drug Reaction………………………………16
(iii) Unexpected Adverse Reaction………………………16
(iv) Serious or Severe Adverse Event……………………16
(v) Serious Adverse Event or Adverse Drug Reaction……..16
(vi) Expected Drug Reaction……………………………….17
(vii) Expected Serious Adverse Event……………………17
(viii) Causality Assessment…………………………………17
7.0 Serious Adverse Event Reporting Procedures…………………..17
7.1 Reporting procedures for all adverse events………………..17
8.0 Statistics……………………………………………………………18

8.1 Description of statistical methods……………………………18
8.2 The number of subjects………………………………………18
8.3 The level of statistical significance…………………………..18
9.0 Monitoring……………………………………………………..19
9.1 Direct Access to Source Data/Documents……………………19
9.2 Quality Control and Quality Assurance Procedures…………19
10.0 Ethics…………………………………………………………..20
10.1 Declaration of Helsinki………………………………………20
10.2 ICH Guidelines for Good Clinical Practice………………….20
10.3 Informed Consent…………………………………………… 20
10.4 Research Ethics Committee………………………………….20
11.0 Confidentiality and Data Handling………………………………..20
11.1Subject Confidentiality……………………………………….20
11.2 Data Handling and Record Keeping…………………………21
12.0 Financing and Insurance…………………………………………..21
13.0 Publication Policy…………………………………………………..21
14.0 References……………………………………………………… 21

Protocol Synopsis

Type of trial:	Pilot trial	
Study Location:		Oxford, UK
Lead Investigators:		A David Smith, Robin Jacoby (OPTIMA, Oxford)<br>Helga Refsum (OPTIMA and Oslo)
Study Design:		Randomised, placebo-controlled (double dummy) with 2 treatment arms (vitamins B6, B12, folic acid; placebo)
Indication:		Mild Cognitive Impairment (MCI), (amnestic-, single non-memory domain-, or multiple domain MCI, Petersen's criteria, 2003)
Objectives:		To determine effect of treatment on effect of treatment on rate of shrinkage of the brain and on memory function; effectiveness of recruitment procedure, proportion responding biochemically to treatment as predicted, acceptability of cognitive tests and within-person variability for future power calculations.
Sample size		350 participants, 175 per treatment arm. Sample size has power to detect differences in biochemical endpoints and rate of brain shrinkage in response to treatment.
Entry criteria	Inclusion	70 years or older<br>MCI<br>TICS-m score and category fluency within defined cut-off scores (see above)<br>Have an informant who knows them well and can be present at visits
	Exclusion	Dementia<br>Treatment with drugs for dementia<br>Diagnosis of active cancer<br>Major stroke within the last 3 months<br>Have a diagnosis of pernicious anaemia<br>Have had some of the small intestine removed at operation<br>Are taking anti-cancer or epileptic drugs or methotrexate<br>Treatment for Vitamin B deficiency (e.g., B12 Injections)<br>Medication exclusion: subjects taking one or more of the following:<br>Pyridoxine (vitamin B6) OVER 3mg per day<br>Folic acid OVER 300 micrograms per day<br>Cobalamin (vitamin B12) OVER 1.5 micrograms per day by mouth
Randomisation criteria		Centralised telephone randomisation 1:1 with full allocation concealment and minimisation (age, gender, baseline cognitive scores)
Recruitment screening		Respondents to advertising will be sent trial information and if interested, then screened with the "telephone interview for cognitive status-modified" (TICS-m), 8min.

Duration of treatment		24 months
Clinic visits		Enrolment, 24 months
Telephone contact		Once every 3 months a) to check compliance, b) ask about adverse reactions c) brief cognitive follow-up
Treatment		TrioBe® Plus tablets (0.8mg folic acid, 0.5mg vitamin B12, 20 mg vitamin B6) Supplied by Recip AB, Solna, Sweden
Control product		Placebo tablets. Supplied by Recip AB, Solna, Sweden
Dosing regimen		Once daily
Route of administration		Oral
Efficacy endpoints	Primary	Clinical: rate of shrinkage of brain assessed by volumetric MRI , change in memory test score. Biochemical: Lowering of blood levels of homocysteine, cystathionine and methylmalonic acid (MMA) in response to treatment
	Secondary	Diagnosis of dementia by DSM IV, IQCODE, change in any of the cognitive test scores
Safety endpoints		Adverse events<br>Vital signs (heart rate, blood pressure)<br>Tingling and numbness of extremities<br>Laboratory parameters (haematology, biochemistry)
Other endpoints		Mortality, drop-out rates, compliance<br>Laboratory parameters (serum folate, B12, B6, holo-transcobalamin, creatinine, TSH, LDL, HDL, cholesterol)
Assessments	Blood levels<br><br>Genetics<br><br>Cognitive assessments, Visits 1 and 2<br><br>Dementia-rating<br><br>Informant interview<br><br>Health scale<br>Depression<br><br>Brain imaging	Homocysteine, cystathionine, MMA, serum folate, B12, B6, holo-transcobalamin, creatinine, TSH, LDL, HDL, cholesterol; all at first visit and at 24-month visit.<br><br>Common gene variants related to ageing, vascular disease and B vitamin status<br><br>MMSE, Hopkins Verbal Learning Test (HVLT)*, paired associates learning (PAL), CLOX, Trailmaking, category fluency, SDMT, Map Search/attention task.<br>*(HVLT by telephone at 3, 6, 12 & 18 months as well). TICS-M by telephone at 15 & 27 months as well.<br><br>Clinical Dementia Rating (CDR) and DSM IV<br><br>Informant Questionnaire on COgnitive Decline in the Elderly (IQCODE)<br><br>EQ-5D<br>Geriatric depression scale (GDS)<br><br>T1 volumetric MRI scan (ca. 15 mins) close to first visit and at 24 month visit for those who volunteer
Statistics		Standard statistical procedures for clinical trials using analysis by randomised treatment

2. CORE PROTOCOL

2.1 STUDY RATIONALE

Every year, about 150,000 of the elderly in the UK (MRC Alpha study) become cognitively impaired. Around half of these will progress to dementia, principally Alzheimer's disease (AD) and AD mixed with cerebrovascular disease. The world faces a growing epidemic of dementia as the population ages: in 2000 there were about 25 million with dementia but by 2030 there will be over 60 million. The burden of dementia upon health care and social security systems and on families is huge: the annual cost of dementia in the UK estimated by the MRC CFAS study is £6.3 billion for 1994 rising to £13.5 billion in 2031 compared with £3.2 billion for stroke, £4 billion for heart disease and £1.2 billion for cancer in 2000.

It is thus urgent that ways are found to reduce the incidence of dementia. The best approach is to identify risk factors for dementia that can be modified by simple and safe interventions. In the proposed trial we target a newly discovered risk factor for dementia, elevated plasma total homocysteine (tHcy). Levels of tHcy can readily be lowered by dietary supplements of three B vitamins: folic acid, B12 and B6. Even a 10% reduction in the conversion of cognitive impairment to dementia would have great impact: it could potentially protect several hundred thousand people world-wide from dementia, which would relieve suffering and eventually save society large sums of money: about $10 billion annually in the USA and £1 billion annually in the UK, [1.2.3.4].

2.2 AIMS AND HYPOTHESES

The purpose is to pilot a trial whose aims would be to answer the questions: • Can the lowering of plasma tHcy levels by administration of folic acid, vitamins B12 and B6 prevent conversion from Mild Cognitive Impairment (MCI) to dementia? • Can the lowering of plasma tHcy levels by administration of these B vitamins slow cognitive decline?

Discussions with experts about the above trial raised a number of critical questions, answers to which would help to optimise the trial design. These questions form the basis of the present proposal.

THE AIM of present pilot trial is to answer practical questions that will improve the design and reduce the costs of a future larger multicentre trial:

- How feasible is it to recruit elderly subjects with cognitive impairment by initial telephone screening?
- Should recruitment be limited to those with MCI who also have elevated tHcy levels?
- What cognitive tests are best tolerated by the subjects and how often should they be carried out?
- What biochemical markers of vitamin status should be measured and how often should they be measured?
- Are levels of these biochemical markers different in the three different categories of cognitive impairment?

- Can treatment with B vitamins slow the rate of shrinkage of the brain in people with memory impairment?
- Is a high dose of vitamin B6 required to achieve maximum lowering of the B6 marker cystathionine?
- What are the annual mortality, dropout rate and compliance in such a cohort and what role do carers play? Another important question, not addressed here, because of the 2 year time limitation, concerns the conversion rate from MCI to dementia; the literature is not consistent about this but new information, some from within our own cohorts, should provide a better estimate by the time the large-scale trial starts.

2.3 SCIENTIFIC BACKGROUND (see reviews by Smith[5] and Morris[6]).

Dementia is a multifactorial disease. Some of the risk factors for dementia are non-genetic and one that has recently been identified is an elevated blood level of tHcy. Elevated tHcy is a well-established risk factor of vascular disease, including stroke. In 1998, the Oxford-based OPTIMA showed that elevated tHcy is also associated with confirmed AD and with vascular dementia and with a more rapid rate of disease progression. It has been estimated that about 16% of incident dementia may be due to raised tHcy levels. The blood levels of tHcy are mainly determined by folate and vitamin B12 status, with a modest contribution from vitamin B6. High levels of tHcy or low levels of either folate or B12 have been associated with an increased risk of developing AD several years later. A low level of vitamin B6 is a risk factor for stroke independent of tHcy and is also associated with cognitive deficit and with AD. [(7,8,9,10,11,12,13)].

The biological plausibility for a link between elevated plasma tHcy levels and dementia is three-fold: homocysteine is neurotoxic and interacts with beta-amyloid[14]; it is toxic to blood vessels and is a risk factor for cerebrovascular disease, which often leads to dementia[15]; and raised tHcy levels are associated with atrophy of the hippocampus in the normal elderly, with brain atrophy assessed many years later, and with more rapid atrophy of the hippocampus in AD patients. Low folate status is also associated with brain atrophy.[16,17,18,19] We will test the hypothesis that those treated with B vitamins will show a slower rate of brain shrinkage than those receiving placebo.

Homocysteine levels in the elderly can be lowered by administration of folic acid, B12 and B6. Thus, if elevated tHcy causes cognitive decline and dementia, it is possible that simple vitamin therapy can prevent this devastating condition. The only reliable way to determine if these vitamins can prevent dementia is to carry out a randomised clinical trial: the planned future large multi-centre trial would test the hypothesis that folic acid and B12 alone, or B6 alone, or all three can slow the conversion of mild cognitive impairment to dementia. Before starting a large trial, more experience with the MCI population and with the proposed intervention will be critical for optimising the trial design. The pilot study proposed here greatly extends our the earlier pilot study involving Oxford and Bristol (VITAL), whose aim was simply to establish that tHcy levels in patients with dementia can be lowered by folic acid and B12 supplements[20]. In VITAL, follow-up time was only 3 months and B6 was not used.

There is uncertainty about the dose of B6 needed because until now there has been no readily available assay for the metabolic marker cystathionine. With our collaborator Refsum, we can answer the latter question.

2.4 TRIAL DESIGN

Target population: people with mild cognitive impairment (MCI) who are 70 or older.

Design: randomised, placebo controlled (double-dummy).

Inclusion criteria: MCI (amnestic, non-amnestic or multiple domain), defined according to standard criteria[21,10]

Trial procedures: *Recruitment screening:* Potential participants (people aged 70 years or older with subjective concerns about memory) will be recruited by media advertisements (newspaper and local radio & TV) seeking those of 70 or over with concerns about their memory. We will also scan the brain by MRI before treatment and then again two years later when the treatment has finished. We can then measure the rate of brain shrinkage in each group. MRI will be voluntary for those who consent to participate in the trial and who don't have a cardiac pacemaker.

Trial interventions: Participants will be separately randomised to one of two groups of 150 each: (1) TrioBe® (0.8 mg folic acid, 0.5 mg vitamin B12, 3 mg vitamin B6); (2) placebo.

Method of randomisation: Centralised telephone randomisation with full allocation concealment and minimisation (age; gender; baseline cognitive scores; MRI consent)

Duration: 3 years: up to 18 months to recruit; 24 months of treatment; 6 months for analysis.

Primary trial design outcomes: Effectiveness of recruitment procedure and of telephone screening, and ratio of recruits to replies; does recruitment in a centre decline over time? Mortality, dropout rate and compliance (tablet counts and biochemical tests); importance of carers in compliance; proportion responding biochemically as predicted; acceptability of the different cognitive tests and within-person variability for future power calculations; need for a second cognitive assessment at 12 months.

Primary imaging, biochemical and haematological outcomes: Rate of shrinkage of brain as assessed by volumetric MRI from baseline to 2-year follow-up. Relationships between type of MCI and *baseline measurements* of factors related to tHcy and vitamin status (including haematological status, blood levels of tHcy; serum folate, B12; B6; methylmalonic acid (MMA); holo-transcobalamin; cystathionine; creatinine); factors related to cognition (thyroid status), important CVD risk factors (eg blood pressure, LDL, HDL cholesterol); If additional funding can be raised, biochemical markers (lipids, glucose, cystatin C etc) and genotypes (e.g. apoE genotype; MTHFR genotype) related to cognition, tHcy or CVD will be done. Proportion of MCI subjects with haematological signs of folate or cobalamin deficiency compared with same age group in same community[22]. Biochemical *endpoints after the intervention* will reveal the extent of the metabolic responses to vitamin treatment (i.e. lowering of levels of tHcy, cystathionine and MMA). Demonstration of the degree of lowering of tHcy levels will inform power calculations for the large trial. The change in cystathionine levels will show if a higher dose of B6 is necessary than present in the triple tablet (TrioBe® with 3mg B6, close to RDA).

Secondary cognitive outcomes: 1. diagnosis of dementia by DSM IV (operationalised) in surviving subjects 2. diagnosis of dementia by IQCODE (3.38 or greater) (all randomised patients – IQCODE can be done with informant after death). 3. Change in any of the cognitive test scores. 4. Changes in CDR.

Sample size: Target: 300 patients: 150 patients to receive TrioBe® (the 3 vitamins) and 150 placebo. Participation in the MRI scans will be voluntary and we anticipate that about 80% of subjects will volunteer. The trial is more than adequately powered for the primary imaging endpoint: a slowing in the rate of shrinkage of the brain of 20% in the treated group. We estimate that for this purpose we need 70 subjects in each group for 90% power at alpha = 0.05. In this study we are not powering for any effect on cognition, which is the main aim of the future large multi-centre trial, but cognitive screening will be done after 24 months to see how well subjects tolerate repeating the tests, and to identify any unwanted or unexpected effects of the intervention. Such a result would be important in the design of the large-scale trial. The trial is not powered to detect differences in mortality or clinical effects between treated and placebo groups, but the trial will help us identify common problems and give an estimate of annual drop-out rate. The sample size for this trial will provide than adequate power to detect plausible differences in the biochemical endpoints in response to treatment.

3. DETAILED STUDY PROCEDURES

3.1 Screening by Telephone

Subjects responding to recruitment advertising will be given a screening number, then telephoned to complete a health screening questionnaire relating to the inclusion and exlusion criteria and asked to complete TICS-M and category fluency. If **TICS-M score is between 17-29** and fit the inclusion and exclusion criteria, subject will be eligible. **For borderline cases**: if TICS-M is > 29 but category fluency < 19 ***or*** TICS-M word recall =/< 10 (/20), they will be eligible. Or if TICS-M is < 17 but category fluency is =/> 19 ***or*** word recall is =/> 10/20, they will be eligible. Then subject will be sent an information pack including the Participant Information Sheet by mail. After a few days, they will be telephoned and will be invited in for enrolment if willing to participate. If subject agrees to participate a study ID will be allocated according to site of investigation: A=Oxford and B=Bristol and a letter will be sent to GP's respondent.

3.2 Inclusion and Exclusion Criteria

Inclusion Criteria

1. 70 years or older. Subjects should have had their 70th birthday by the date of Visit 1.
2. MCI (according to TICS-M score)
3. No dementia diagnosis.
4. Have an informant who knows them well enough to complete the IQCODE and the informant CDR and can be present at visits 1 and 2.
5. Has not taken part in another drug study in the past 3 months and is not enrolled concurrently in another drug study (unless with agreement by PI of both studies).
6. On questioning, has adequate hearing and vision to complete study assessments.**

Exclusion Criteria

1. Have a diagnosis of dementia
2. Are on treatment with drugs for dementia
3. Have a diagnosis of active cancer (past history of cancer, inactive or not on treatment to be discussed with PI)
4. Have had a major stroke within the last 3 months
5. Have a diagnosis of pernicious anaemia
6. Have had some of the small intestine removed at operation
7. Are taking anti-cancer or epileptic drugs or methotrexate
8. Are on treatment for Vitamin B deficiency (e.g., B12 Injections)
9. Medication exclusion: subjects taking one or more of the following:
    - Pyridoxine (vitamin B6) OVER 3mg per day
    - Folic acid OVER 300 micrograms per day
    - Cobalamin (vitamin B12) OVER 1.5 micrograms per day by mouth
10. Have any other medical condition(s) that the PI decides are unacceptable for study inclusion.

** See section on home Visits.

3.3 Visit 1 – MCI Classification and Enrolment Visit

Trial information will be further discussed, and questions answered and informed consent taken from subject and informant with signatures.

Check that subject fits all inclusion/exclusion criteria.

Verify all concomitant medication
Document demographic details: age, gender, education level, race.
Brief medical history including question on tingling and numbness of extremities
Geriatric Depression Scale,
EQ-5D and IQCODE (informant)
Vital signs (BP and heart rate)
Urine sample
Blood sample/s
Simple vibration sensitivity test
MMSE
Complete enrolment and assign central telephone randomisation number and pack number
Dispense medication
Send off bloods
Complete ID log for enrolled subjects
Book an MRI scan (for those who volunteer)

MCI status assessment: CDR (subject and informant), neuropsychology battery (see Table, subject only). If any 1 score on neuropsychological test battery is 1.5 SD below age related norms, subject will be classified as:-
i) MCI-amnestic, ii) single, non-memory domain MCI, iii) multiple (non-amnestic) domain MCI iv) Amnestic-multiple MCI.

DSM-IV: Subject will be classified as MCI (0.5) if either CDR = 0.5 and/or neuropsychological battery classifies them as MCI. If CDR=0 and if no neuropsychology test score is 1.5 SD below the age-related norms, then subject will be classified as Normal or 0 on DSM=IV.

3.4 First MRI scan visit

Volunteers who do not have cardiac pacemaker or other metal implant (pacemaker wires, metal fragments – especially near eyes or metal injury), limb or joint prosthesis, severe claustrophobia or serious medical conditions e.g. fits, serious heart condition will undergo a T1 volumetric MRI scan that should last about 15 minutes. This is a research scan only, not a diagnostic scan, and will not contribute to the diagnostic classification at baseline or at Visit 2.

3.5 Treatment

Start of treatment on the day after the MRI scan or, for those not having a scan, the day after randomisation. Treatment is either TrioBe® Plus tablets (0.8mg folic acid, 0.5mg vitamin B12, 20 mg vitamin B6) or placebo.

3.6 3-month Telephone Follow-up

Nurse to report any changes in well-being or concomitant medication
Confirm compliance
HVLT version 2
The date of the 3-monthly follow-ups will be determined from the date subjects commence treatment, not from Visit 1 date. There will be a window of 6 weeks (2 weeks before and 4-weeks after follow-up date) in which to contact subject for follow-up.

3.7 6-month Telephone Follow-up
Report any changes in well-being or concomitant medication
Confirm compliance
HVLT version 3

3.8 9-month Telephone Follow-up
Report any changes in well-being or concomitant medication
Confirm compliance
*To be dropped (Oct 2006).

3.9 12-month Telephone Follow-up
Report any changes in well-being, including question on tingling and numbness in extremities or concomitant medication. If there is a change from Visit 1 in tingling or numbness, refer to GP (?peripheral neuropathy). Nurse to follow-up with GP in one month.
Confirm compliance
HVLT version 4

3.10 15-month Telephone Follow-up
Report any changes in well-being or concomitant medication
Confirm compliance
TICS-M

3.11 18-month Telephone Follow-up
Report any changes in well-being or concomitant medication
Confirm compliance
HVLT version 5

3.12 21-month Telephone Follow-up
Report any changes in well-being or concomitant medication
Confirm compliance
*to be dropped (Oct 06)

3.13 Visit 2 - 24-month follow-up visit

CDR (informant and subject)
Neuropsychology battery (See Table)
IQCODE (informant)

Medical history update
Simple vibration sensitivity test
Follow up questionnaire: changes in well-being including question on tingling and numbness in extremities.
GDS
EQ-5D
MMSE
DSM IV

Blood and urine
BP &HR
Compliance
Adverse effects

3.14 Second MRI scan visit

Volunteers who had an initial MRI scan will undergo a second T1 volumetric MRI scan that should last about 15 minutes.

3.15 Post-study follow-up by telephone (approx. 27 months)

Repeat of TICS-M memory test.
Ask about any changes in well-being

3.16**Home visits

To increase recruitment, if subject has poor hearing at screening by telephone, or reports development of poor hearing during the study, telephone follow-ups will be replaced by home visits.

4.0 MEASUREMENTS AND DATA COLLECTION.

4.1 Medical
Demographic information
Medical History
Medication list
Blood pressure
Urine dipstix
Heart Rate
Weight
Height
Blood collection
DSM-IV
EQ-5D (Health Questionnaire)

4.2 Biochemical
Homocysteine, cystathionine, MMA, serum folate, B12, B6, holo-transcobalamin, creatinine, TSH, LDL, HDL, cholesterol; all at first visit and at 24-month visit.

4.3 Genetic
Common gene variants related to ageing, vascular disease and B vitamin status

4.4 Cognitive
Mini Mental State Examination (MMSE), Hopkins Verbal Learning Test (HVLT)*, CANTAB Paired Associates Learning (PAL), CANTAB Spatial Recognition, CLOX (clock drawing task), Trail- making A&B, category fluency (Fruits and vegetables, 1 minute), Symbol Digits Modalities Test (SDMT), Map Search (Everyday Attention Battery).
*(HVLT by telephone at 3, 6, 12 & 18 months as well). TICS-M by telephone at 15 & 27 months as well.

4.5 Neuropsychological

Clinical Dementia Rating (CDR)
Geriatric depression scale (GDS)
Informant Questionnaire on COgnitive Decline in the Elderly (IQCODE)

4.6 Neuro-imaging

T1 volumetric MRI scan (ca. 15 mins) close to first visit and at 24 month visit for those who volunteer

Assessment	Screening	Enrolment	3-month FU	6-month FU	9-month FU	12-month FU	15-month FU	18-month FU	21-month FU	24-month FU	27-month FU	Nurse	NPsychol	Time
	Tel	**Visit 1**	Tel	Tel	Tel	Tel	Tel	Tel	Tel	**Visit 2**	Tel			(min)
TICS-M	X						X				X	x		10
Informed consent		X										x		20
		X										x		20
Medical History		X								X		x		20
Vibration test		X								X		x		10
GDS		X								X		x		5
EQ-5D		X								X		x		5
CDR		X								X			x	60
HVLT		X	X	X		X		X		X			x	10
SDMT		X								X			x	3
Category Fluency		X								X			x	2
Paired Associate Learning		X								X			x	10
Graded Naming		X								X			x	5
CLOX		X								X			x	4
Trailmaking		X								X			x	4
Attention Task/Mapsearch		X								X				5
BP		X								X		x		
Urine dipstix		X								X		x		
Heart Rate		X								X		x		30
Weight		X										x		
Height		X										x		
Bloods		X								X		x		
DSMIII-R										X		x		15
IQCODE										X		x		5
Randomisation		X										x		10
Dispensing of Meds		X										x		15
MRI scan		X								X				

(voluntary)														
Compliance			X	X	X	X	X	X	X	X		x		5
Adverse effects			X	X	X	X	X	X	X	X		x		5
Concomitant Medication		X	X	X	X	X	X	X	X	X		x		5

6.0 ASSESSMENT OF SAFETY

6.1VitaminSafety

A search of the medical literature has shown that there are no reports of harmful effects of the vitamins to be used in the doses proposed. A recent study (Toole et al. JAMA 2004, 291:565-575) administered similar or higher doses of these vitamins to nearly 2,000 participants for two years and found no adverse effects.

In addition to the safety assessments carried out at pre study screen and post study clinical check, which are described within Sections 3, the following assessments will be carried out on visits to the clinic/telephone follow-ups:

Visits 1 and 2. Vibration test and Tingling and numbness questions to rule out peripheral neuropathy. On 12-month telephone follow-up any changes in well-being, including question on tingling and numbness in extremities. If there is a change from Visit 1 in tingling or numbness, refer to GP (?peripheral neuropathy). Nurse to follow-up with GP in one month.

6.2 Definitions

(i) Adverse Event (AE)

An AE or adverse experience is:

Any untoward medical occurrence in a patient or clinical investigation subject administered a medicinal product, which does not necessarily have to have a causal relationship with this treatment (the study medication).

An AE can therefore be any unfavourable and unintended sign (including an abnormal laboratory finding), symptom or disease temporally associated with the use of the study medication, whether or not considered related to the study medication.

(ii) Adverse Drug Reaction (ADR)

All untoward and unintended responses to a medicinal product related to any dose.

The phrase "responses to a medicinal products" means that a causal relationship between a study medication and an AE is at least a reasonable possibility, i.e., the relationship cannot be ruled out.

(iii) Unexpected Adverse Reaction

An adverse reaction, the nature or severity of which is not consistent with the applicable product information (eg. Investigator’s brochure or summary of product characteristics).

(iv) Serious or Severe Adverse Events

To ensure no confusion or misunderstanding of the difference between the terms "serious" and "severe", which are not synonymous, the following note of clarification is provided:

The term "severe" is often used to describe the intensity (severity) of a specific event (as in mild, moderate, or severe myocardial infarction); the event itself, however, may be of relatively minor medical significance (such as severe headache). This is not the same as "serious," which is based on patient/event outcome or action criteria usually associated with events that pose a threat to a patient's life or functioning. Seriousness (not severity) serves as a guide for defining regulatory reporting obligations.

(v) Serious Adverse Event or Adverse Drug Reaction

A serious adverse event (experience) or reaction is any untoward medical occurrence that at any dose:

- Results in death,
- Is life-threatening,

NOTE: The term "life-threatening" in the definition of "serious" refers to an event in which the patient was at risk of death at the time of the event; it does not refer to an event which hypothetically might have caused death if it were more severe.

- Requires inpatient hospitalisation or prolongation of existing hospitalisation,
- Results in persistent or significant disability/incapacity, or

- Is a congenital anomaly/birth defect.

Medical and scientific judgment should be exercised in deciding whether an adverse event is serious in other situations.

(vi) Expected Adverse Drug Reactions

We do not expect any side-effects from the treatment. The combination of B vitamins in TrioBe® Plus has been used in several large trials on heart disease and stroke in many thousands of people without any reported harmful effects. But outside these studies it has been reported that in rare cases some people develop a skin rash like acne, which goes away once the tablets are stopped.

(vii) Expected Serious Adverse Events

None expected.

(viii) Causality Assessment

All cases judged by either the reporting medically qualified professional or the sponsor as having a reasonable suspected causal relationship to the study medication qualify as ADRs.

7.0 Serious Adverse Event Reporting Procedures

All SAEs, except those that do not require immediate reporting, must be reported to the sponsor/PI within one working day of discovery or notification of the event. All SAE information must be recorded on an SAE forms and faxed to the sponsor/PI. Additional information received for a case (follow-up or corrections to the original case) need to be detailed on a new SAE form and faxed to the sponsor.

The sponsor/PI will report all suspected adverse reactions which are both serious and unexpected (SUSARs) to the Competent Authorities (MHRA) and the Research Ethics Committee concerned. Fatal or life-threatening SUSARs must be reported within 7 days and all other SUSARs within 15 days. The sponsor/PI will also inform all investigators concerned of relevant information about SUSARs that could adversely affect the safety of subjects.

In addition to the expedited reporting above, the sponsor/PI shall submit once a year throughout the clinical trial or on request a safety report to the Competent Authority and Ethics Committee.

7.1 Reporting Procedures for All Adverse Events

All AEs occurring during the study observed by the investigator or reported by the patient, whether or not attributed to study medication, will be reported on the CRF. AEs considered related to the study medication by the investigator or the sponsor will be followed until resolution or the event is considered stable. The following attributes must be assigned by the investigator: description, date of onset and resolution date, severity, assessment of relatedness to study medication, other suspect drug or device and action taken. The investigator may be asked to provide follow-up information.

All related AEs that result in a patient's withdrawal from the study or are present at the end of the study, should be followed up until a satisfactory resolution occurs.

All deaths occurring on study must be reported to the Sponsor/PI. These include deaths within 30 days of the final dose of study medication and deaths up to the last formal follow-up observational period, whichever is longer. For all deaths, available autopsy reports and relevant medical reports will be made available for reporting to the relevant authorities.

The investigator should notify the REC of SUSARs occurring at the centre in accordance with local procedures.

It will be left to the investigator's clinical judgment whether or not an AE is of sufficient severity to require the patient's removal from treatment. A patient may also voluntarily withdraw from treatment due to what he or she perceives as an intolerable AE, further details of withdrawal are presented in Section 10. If either of these occurs, the patient must undergo an end of study assessment and be given appropriate care under medical supervision until symptoms cease or the condition becomes stable.

The severity of events will be assessed on the following scale: 1 = mild, 2 = moderate, 3 = severe. The relationship of AEs to the study medication will be assessed according to the definition provide in Section.

8.0 STATISTICS

8.1 Description of Statistical Methods

Standard statistical procedures for clinical trials using analysis by randomised treatment. For the analysis of MRI data we will use per protocol analysis since data from the second MRI scan is required in order to determine the rate of atrophy of the brain. Otherwise, we will use intention to treat analysis and the appropriate advanced statistical tools for mixed effectsmethods.

8.2 The Number of Subjects

The estimated power is at least 90% at alpha = 0.05 two-tailed (unpaired t test of means) to detect a 20% effect of treatment on the rate of shrinkage of the brain, based upon our existing data using the same MRI procedures in a group of elderly with MCI. In 49 with MCI, rate of shrinkage was 0.74 ml/y SD = 0.27. To detect a 20% reduction in rate, we need 70 subjects per group for 90% power and 80 per group for 95% power. If the effect size is 25%, we need 50 or 60 per group, respectively. Thus, if 25% of the subjects die or drop out during the treatment period we will still have sufficient power to detect a 20% effect. Similar values have been reported by Jack et al. Neurology, 62:591 (2004). The trial is not powered to detect differences in mortality or clinical/neuropsychological differences between treated and placebo groups, but the trial will help us identify common problems and give an estimate of annual drop-out rate. The sample size for this trial was chosen to have more than adequate power to detect plausible differences in the biochemical endpoints in response to treatment [Clarke et al 1998 BMJ 316, 894].

8.3 The Level of Statistical Significance

VITACOG Power estimates

Anticipated SD of each group (assume equal): 0.27, Alpha=0.05, two-tailed.

N per group	99% Power	95% Power	90% Power	85% Power	80% Power
3	1.20	1.01	0.91	0.84	0.79
4	0.97	0.81	0.73	0.68	0.63
5	0.83	0.70	0.63	0.58	0.54
6	0.74	0.62	0.56	0.52	0.48
7	0.67	0.57	0.51	0.47	0.44
8	0.62	0.52	0.47	0.44	0.41
9	0.58	0.49	0.44	0.41	0.38
10	0.55	0.46	0.42	0.38	0.36
12	0.50	0.42	0.38	0.35	0.32
14	0.46	0.38	0.34	0.32	0.30
16	0.42	0.36	0.32	0.30	0.28
18	0.40	0.33	0.30	0.28	0.26
20	0.38	0.32	0.28	0.26	0.25
25	0.33	0.28	0.25	0.23	0.22
30	0.30	0.26	0.23	0.21	0.20
35	0.28	0.24	0.21	0.20	0.18
40	0.26	0.22	0.20	0.18	0.17
50	0.23	0.20	0.18	0.16	0.15
60	0.21	0.18	0.16	0.15	0.14
70	0.20	0.17	0.15	0.14	0.13
80	0.18	0.15	0.14	0.13	0.12
90	0.17	0.15	0.13	0.12	0.11
100	0.16	0.14	0.12	0.12	0.11
125	0.15	0.12	0.11	0.10	0.10
150	0.13	0.11	0.10	0.09	0.09
175	0.12	0.10	0.09	0.09	0.08
200	0.12	0.10	0.09	0.08	0.08
250	0.10	0.09	0.08	0.07	0.07
300	0.09	0.08	0.07	0.07	0.06

Highlight shows 90% power estimate for a 20% reduction in rate of shrinkage (0.148%)

9.0 MONITORING

9.1 Direct Access to Source Data/Documents

The investigator(s)/institution(s) will permit trial-related monitoring, audits, REC review, and regulatory inspection(s), providing direct access to source data/documents to authorised auditors and monitors.

9.2 Quality Control and Quality Assurance Procedures

Data will be evaluated for compliance with the protocol and accuracy in relation to source documents. The study will be conducted in accordance with procedures identified in the protocol. SOPs will be used at all clinical and laboratory sites. Regular monitoring will be performed according to ICH GCP. Following written standard operating procedures, the monitors will verify that the clinical trial is conducted and data are generated, documented and reported in compliance with the protocol, GCP and the applicable regulatory requirements. The investigator site will provide direct access to all trial related source data/documents and reports for the purpose of monitoring and auditing by the sponsor, and inspection by local and regulatory authorities.

10.0. Ethics

10.1 Declaration of Helsinki

The Investigator will ensure that this study is conducted in full conformity with the current revision of the Declaration of Helsinki (last amended October 2000, with additional footnotes added 2002 and 2004).

10.2 ICH Guidelines for Good Clinical Practice

The Investigator will ensure that this study is conducted in full conformity with relevant regulations and with the ICH Guidelines for Good Clinical Practice (CPMP/ICH/135/95) July 1996.

10.3 Informed Consent

Written and verbal versions of Informed consent will be presented to the subject detailing no less than: the exact nature of the study; the implications and constraints of the protocol; the known side effects and any risks involved in taking part. It will be clearly stated that the patient/subject is free to withdraw from the study at any time for any reason without prejudice to future care, and with no obligation to give the reason for withdrawal.

The patient/subject will be allowed as much time as wished to consider the information, and the opportunity to question the Principal Investigator, their GP or other independent parties to decide whether they will participate in the study. Written Informed Consent will then be obtained by means of subject dated signature, signature of the person who presented informed consent and, if different, the Principal Investigator (or named Co-Investigator). A copy of the signed Informed Consent will be given to the subject. The original signed form will be retained at the study site.

10.4 Research Ethics Committee

A copy of the protocol, proposed informed consent form, other written patient/subject information and any proposed advertising material will be submitted to an Research Ethics Committee (REC) for written approval.

The Investigator will submit and, where necessary, obtain approval from the REC for all subsequent protocol amendments and changes to the informed consent document.

The Investigator will notify deviations from the protocol or SAEs occurring at the site to the sponsor and will notify the REC of these in accordance with local procedures.

11.0 Confidentiality and Data Handling

11.1 Subject Confidentiality

The Investigator will ensure that the subject's anonymity is maintained. The subject will be identified only by initials and a subject ID number on the CRF. All documents will be stored securely and kept in strict confidence in compliance with the Data Protection Act.

All information collected during the course of the study will be kept strictly confidential and recorded in a way that fulfils the Data Protection Act.

The subject will be identified by a study specific subject number and/or code. The name and any other identifying detail will NOT be included in any study data electronic file. Any information that leaves the

University will subject names and addresses removed so that participants cannot be recognised from it. The results from any later analysis of blood and urine samples will likewise be kept strictly confidential.Participants' names will not be used in any publication.

11.2 Data Handling and Record Keeping

All study data (CRF, diary, other assessment data) will be entered on a SQL database, with data entry screens programmed in PHP/Apache. The data will be backed up twice daily to the University HFS storage, so there are always 2 copies only a few hours part in 2 separate locations. Once the data is complete, the database will be backed up onto CD.

12.0 Financing and Insurance

Deciding not to take part in the study or to withdraw from the study will not affect participants' treatment in the National Health Service

There are no special arrangements for compensation in the unlikely event of participants suffering any study related injury or side effect of treatment. They would retain the same legal rights as any other patient in the National Health Service and the University as sponsor maintains professional and 'no-fault' insurance that will apply to this study.

The Medical Research Council has awarded a special grant for this trial. OPTIMA has also received financial support from charitable foundations, including the Charles Wolfson Charitable Trust and the Smith Charity.

13.0 Publication Policy

The Investigators will co-ordinate dissemination of data from this study. All publications (e.g., manuscripts, abstracts, oral/slide presentations, book chapters) based on this study will be submitted to peer reviewed journals before release.

Key audiences will be funding bodies for the large-scale trial.

14.0 REFERENCES

1.Copeland, J. R. M., McCracken, C. F. M. et al. Undifferentiated dementia, Alzheimer's disease and vascular dementia: age- and gender-related incidence in Liverpool - The MRC-ALPHA Study. Brit J Psychiat 175, 433-438 (1999).

2.Wimo, A., Winblad, B., Aguero-Torres, H. & von Strauss, E. The magnitude of dementia occurrence in the world. Alzheimer Dis Assoc Disord 17, 63-7 (2003). 3.McNamee, P., Bond, J. & Buck, D. Costs of dementia in England and Wales in the 21st century. Br J Psychiatry 179, 261-6. (2001).

4.Lowin, A., Knapp, M. & McCrone, P. Alzheimer's disease in the UK: comparative evidence on cost of illness and volume of health services research funding. Int J Geriatr Psychiatry 16, 1143-8. (2001).

5.Smith, A. D. Homocysteine, B vitamins, and cognitive deficit in the elderly. Am J Clin Nutr 75, 785-6. (2002).

6.Morris, M. S. Homocysteine and Alzheimer's disease. Lancet Neurol 2, 425-8 (2003).

7.Refsum, H., Ueland, P., Nygard, O. & Vollset, S. E. Homocysteine and cardiovascular disease. Annu Rev Med 49, 31-62 (1998).

8.Clarke, R., Smith, A. D. et al. Folate, vitamin B12, and serum total homocysteine levels in confirmed Alzheimer disease. Arch Neurol 55, 1449-55 (1998).

9.Seshadri, S., Beiser, A. et al. Plasma homocysteine as a risk factor for dementia and Alzheimer's disease. N Engl J Med 346, 476-483. (2002).

10.Clarke, R., Frost, C., Leroy, V. & Collins, R. Lowering blood homocysteine with folic acid based supplements: meta-analysis of randomised trials. Br Med J 316, 894-898 (1998).

11.Wang, H. X., Wahlin, A. et al. Vitamin B12 and folate in relation to the development of Alzheimer's disease. Neurology 56, 1188-94. (2001).

12.Riggs, K. M., Spiro, A., Tucker, K. & Rush, D. Relations of vitamin B-12, vitamin B-6, folate, and homocysteine to cognitive performance in the Normative Aging Study. Am. J. Clin. Nutr. 63, 306-314 (1996).

13.Miller, J. W., Green, R., Mungas, D. M., Reed, B. R. & Jagust, W. J. Homocysteine, vitamin B, and vascular disease in AD patients. Neurology 58, 1471-5. (2002).

14.Kruman, II, Kumaravel, T. S. et al. Folic Acid deficiency and homocysteine impair DNA repair in hippocampal neurons and sensitize them to amyloid toxicity in experimental models of Alzheimer's disease. J Neurosci 22, 1752-62. (2002).

15.Sachdev, P. S., Valenzuela, M. J. et al. Homocysteine as a risk factor for cognitive impairment in stroke patients. Dement Geriatr Cogn Disord 15, 155-62. (2003).

16.Williams, J. H., Pereira, E. A., Budge, M. M. & Bradley, K. M. Minimal hippocampal width relates to plasma homocysteine in community-dwelling older people. Age Ageing 31, 440-4. (2002).
17.Den Heijer, T., Vermeer, S. E. et al. Homocysteine and brain atrophy on MRI of non-demented elderly. Brain 126, 170-175. (2003).
18.Seshadri, S., Wolf, P. A. et al. Elevated Plasma Homocysteine Levels Are Associated with Subclinical Brain Injury in Healthy Adults: Relation to Silent Cerebral Infarcts, White Matter Hyperintensity and MRI Brain Volume in the Framingham Offspring. AAN Abstracts, Abstract S08.006 (2003).
19.Snowdon, D. A., Tully, C. L., Smith, C. D., Riley, K. P. & Markesbery, W. R. Serum folate and the severity of atrophy of the neocortex in Alzheimer disease: findings from the Nun study. Am J Clin Nutr 71, 993-998 (2000).
20.VITAL. Effect of vitamins and aspirin on markers of platelet activation, oxidative stress and homocysteine in people at high risk of dementia. J Intern Med 254, 67-75 (2003).
21.Petersen, R. C., Stevens, J. C. et al. Practice parameter: early detection of dementia: mild cognitive impairment (an evidence-based review). Report of the Quality Standards Subcommittee of the American Academy of Neurology. Neurology 56, 1133-42. (2001). 22.Clarke, R., Refsum, H. et al. Screening for vitamin B-12 and folate deficiency in older persons. Am J Clin Nutr 77, 1241-7. (2003).
23.Aisen, P. S., Egelko, S. et al. A pilot study of vitamins to lower plasma homocysteine levels in Alzheimer disease. Am J Geriatr Psychiatry 11, 246-9. (2003).
24.De Jager, C. A., Budge, M. M. & Clarke, R. Utility of TICS-M for the assessment of cognitive function in older adults. Int J Geriatr Psychiatry 18, 318-24. (2003).
